# Brief Report: Exercise and Blood Pressure in Older Adults—An Updated Look

**DOI:** 10.1155/2018/6548659

**Published:** 2018-10-29

**Authors:** George A. Kelley, Kristi S. Kelley

**Affiliations:** ^1^Meta-Analytic Research Group, School of Public Health, Department of Biostatistics, Robert C. Byrd Health Sciences Center, West Virginia University, Morgantown, WV, USA; ^2^School of Public Health, Department of Biostatistics, Robert C. Byrd Health Sciences Center, West Virginia University, P.O. Box 9190, Morgantown, WV, USA

## Abstract

**Background/Objectives:**

Raised blood pressure is a major problem in older adults. Using a random-effects model, a recent meta-analysis reported statistically significant reductions in both resting systolic blood pressure (SBP) and diastolic blood pressure (DBP) as a result of aerobic, resistance, and combined aerobic and resistance exercise in adults ≥65 years. To provide more objective information regarding this nonpharmacologic approach, this study applied more robust methods to this data.

**Design:**

Meta-analysis of 41 randomized controlled trials representing 96 groups (52 exercise; 44 control).

**Setting:**

Any location where a randomized controlled trial could be conducted.

**Participants:**

Adults ≥65 years.

**Intervention:**

Trials ≥2 weeks that included aerobic, resistance, and/or combined aerobic and resistance exercise as the intervention.

**Measurements:**

The recently developed inverse heterogeneity model (IVhet) was used to pool findings and the Doi plot was used to examine for small-study effects. Absolute and relative differences between the IVhet and random-effects model were also calculated. Data were reported using the mean difference (exercise minus control) with nonoverlapping 95% confidence intervals considered statistically significant.

**Results:**

Statistically significant reductions in resting blood pressure were found as a result of aerobic exercise (SBP, -4.7 mmHg, 95% CI, -7.7 to -1.8; DBP, -2.0 mmHg, 95% CI -3.13 to -0.9), SBP but not DBP for resistance training (SBP, -7.0 mmHg, 95% CI, -10.5 to -3.4; DBP, -1.2 mmHg, 95% CI -2.7 to 0.3), and both SBP and DBP for combined aerobic and resistance training (SBP, -5.5 mmHg, 95% CI, -8.3 to -2.7; DBP, -3.7 mmHg, 95% CI -4.8 to -2.7).

**Conclusions:**

Exclusive of changes in DBP congruent with resistance training, exercise (aerobic, resistance, and combined aerobic and resistance) reduces resting SBP and DBP in older adults. These findings have practical implications when considering exercise for the prevention and treatment of raised blood pressure in older adults.

## 1. Introduction

Raised blood pressure, defined as a resting systolic blood pressure (SBP) ≥140 mmHg and/or diastolic blood pressure (DBP) ≥90 mmHg, is a major risk factor for cardiovascular morbidity and mortality [1] and is especially prevalent among older adults [2]. In adults 60 years of age and older, the worldwide prevalence of raised blood pressure has been reported to be 44.1%, 47.8%, 50.3%, 51,7%, 51.6%, and 50.2%, respectively, in men 60-64, 65-69, 70-74, 75-79, 80-84, and 85+ years of age [2]. For women, prevalence rates have been estimated to be 43.7%, 48.9%, 53.2%, 56.2%, 57.3%, and 65.9% in those 60-64, 65-69, 70-74, 75-79, 80-84, and 85+ years of age [2]. Most notably, the number of deaths worldwide from raised blood pressure has been estimated to be 7.5 million (12.8% of the total of all deaths) [3]. One recommended nonpharmacologic intervention for the prevention and treatment of elevated resting SBP and DBP is exercise [1]. Using a random-effects model, a recent meta-analysis by Herrod et al. (2018) reported statistically significant reductions in both resting systolic blood pressure (SBP) and diastolic blood pressure (DBP) as a result of aerobic, resistance, and combined aerobic and resistance exercise in adults with a mean age of 65 years and older [4]. However, a more robust method, the inverse heterogeneity model (IVhet), has recently been developed for pooling the results for a meta-analysis [5]. Providing more robust estimates is important for providing the best evidence regarding the effects of exercise on resting SBP and DBP in older adults. Therefore, the purpose of this brief communication was to apply the IVhet model to these previous meta-analytic findings.

## 2. Materials and Methods

### 2.1. Data Source

Data for this brief communication were derived from a recent systematic review with meta-analysis focused on the effects of exercise (aerobic, resistance training, or both) on any level of resting SBP and DBP in older adults, details of which have been described elsewhere [4]. Briefly, 41 randomized controlled trials representing 96 groups (52 exercise; 44 control) in adult humans with a mean age of 65 years and older and in which exercise was performed were included [4]. The length of the interventions ranged from 8 to 52 weeks ($\overset{-}{X}\;\pm$ SD = 19 ± 10, median = 16), frequency from 1 to 5 times per week ($\overset{-}{X}\;\pm$ SD = 3 ± 1, median = 3), and duration from 25 to 120 minutes per session ($\overset{-}{X}\;\pm$ SD = 51 ± 21, median = 53). For those studies that included resistance exercise, the number of sets ranged from 1 to 3 ($\overset{-}{X}\;\pm$ SD = 2 ± 1, median = 2), repetitions from 8 to 30 ($\overset{-}{X}\;\pm$ SD = 15 ± 10, median = 12), and number of exercises from 1 to 9 ($\overset{-}{X}\;\pm$ SD = 6 ± 2, median = 7). Intensity of the exercise interventions was usually performed at a moderate to high level. Mean baseline resting SBP ranged from 120.0 to 184.0 mmHg in the exercise groups ($\overset{-}{X}\;\pm$ SD = 139.4 ± 12.0, median = 138.0) and 121.0 to 182.0 mmHg in the control groups ($\overset{-}{X}\;\pm$ SD = 138.2 ± 10.7, median = 136.0). For resting DBP, values ranged from 61.0 to 90.0 mmHg in the exercise groups ($\overset{-}{X}\;\pm$ SD = 77.9 ± 5.8, median = 78.0) and 68.0 to 89.0 in the control groups ($\overset{-}{X}\;\pm$ SD = 78.0 ± 5.3, median = 78.0).

### 2.2. Effect Size Calculations

The effect sizes for the current study were derived using the original metric (mmHg) from previously reported exercise minus control group changes in resting SBP and DBP along with their 95% confidence intervals [4].

### 2.3. Effect Size Pooling

Changes in resting SBP and DBP according to type of intervention (aerobic, resistance training, or both) were pooled using the recently developed IVhet model [5]. Briefly, the IVhet model is a quasilikelihood model that is computed by calculating weights that sum to 1 from each study, pooling effect sizes from all studies and then calculating the variance of the pooled effect size [5]. Two-tailed z-alpha values ≤0.05 were considered statistically significant. Heterogeneity and inconsistency for each pooled outcome were estimated using the Q [6] and* I*^*2*^ statistics [7], respectively. An alpha level of <0.10 for Q was considered to represent statistically significant heterogeneity while inconsistency was categorized as very low (<25%), low (25% to <50%), moderate (50% to <75%), or large (≥75%) [7]. Small-study effects (publication bias, etc.) were examined using the recently developed Doi plot [8], an approach considered to provide more robust estimates of asymmetry, i.e., small-study effects, than previously developed and recommended approaches [9]. This plot also includes a quantitative measure, the Luis Furuya-Kanamori (LFK) index for determining asymmetry [8]. Values ± 1 are considered to represent no asymmetry, values greater than ± 1 but within ± 2, minor asymmetry, and values greater than ± 2, major asymmetry [8]. Sensitivity of changes in resting SBP and DBP according to type of training was examined by deleting each result from each model once. All analyses were performed using Meta XL, version 5.3 (Epigear International, Canberra, Australia).

## 3. Results

**Figure 1:**
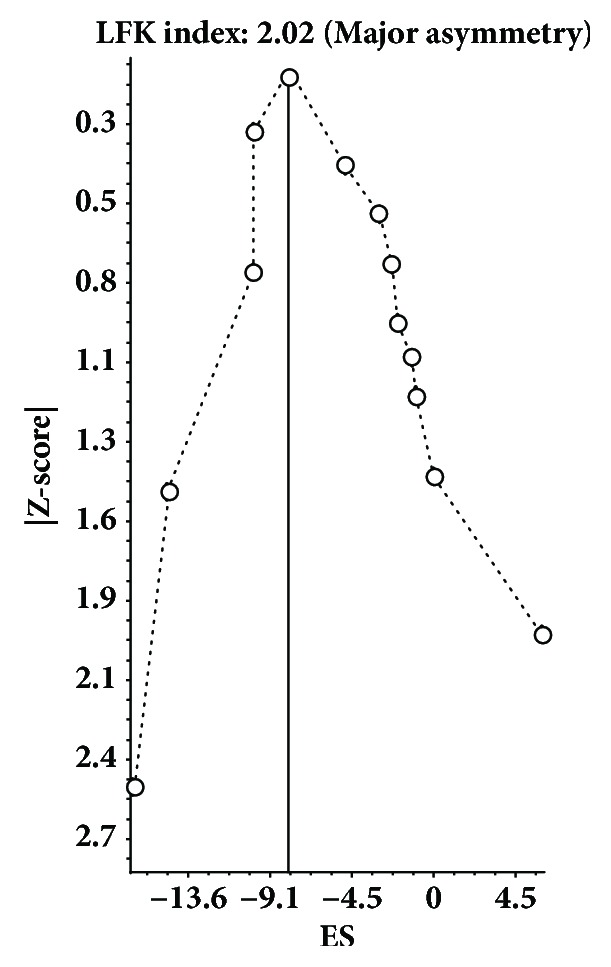
Doi plot and LFK index for small-study effects based on changes in resting SBP as a result of resistance exercise.

A summary of changes in resting SBP and DBP using the IVhet model is shown in Table 1, study-level results are shown in Supplementary Files 1-6, and Doi plots for asymmetry, i.e., small-study effects, are shown in Table 2, Figure 1, and Supplementary Files 7-11. As can be seen, statistically significant reductions in both SBP and DBP were found as a result of aerobic exercise. Statistically significant heterogeneity was observed along with a large and moderate amount of inconsistency for SBP and DBP, respectively (Table 1; Supplementary Files 1 and 2). No asymmetry was observed for either SBP (LFK index = -0.32, Table 2; Supplementary File 7) or DBP (LFK index = -0.89, Table 2; Supplementary File 8). With each result deleted from the model once, changes in resting SBP ranged from -5.8 to -4.0 mmHg while changes in DBP ranged from -2.3 to -1.8 mmHg. For resistance training, statistically significant reductions were found for SBP but not DBP (Table 1; Supplementary Files 3 and 4). Statistically significant heterogeneity and moderate inconsistency were observed for both resting SBP and DBP. Major asymmetry was observed for both SBP (LFK index = 2.02, Table 2; Figure 1) and DBP (LFK index = -3.21, Table 2; Supplementary File 9). With each study deleted from the model once, changes ranged from -7.6 to -6.0 mmHg for SBP and -2.3 to -1.0 mmHg for DBP. For those groups who participated in both aerobic and resistance training, statistically significant reductions were found for both resting SBP and DBP. Statistically significant heterogeneity were observed for both SBP and DBP while a moderate and low amount of inconsistency was observed for SBP and DBP, respectively (Table 1; Supplementary Files 5 and 6). No asymmetry was observed for either SBP (LFK index = 0.06, Table 2; Supplementary File 10) or DBP (LFK index = 0.84, Table 2; Supplementary File 11). With each study deleted from the model once, changes ranged from -6.0 to -5.0 mmHg for SBP and -3.9 to -3.1 mmHg for DBP.

When compared to meta-analytic results from the original study using the random-effects model [4], findings from four of the six mean differences (66.7%) in resting SBP and DBP were smaller, ranging from -0.82 to -0.19 mmHg (6.1% to 41.0%) while all six 95% CI were wider, ranging from 0.24 to 1.56 mmHg (11.5% to 36.8%).

## 4. Discussion

Using a more robust model, the findings of the current brief report suggest that, with the exception of changes in DBP as a result of resistance training, exercise (aerobic, resistance, and combined aerobic and resistance) reduces resting SBP and DBP in older adults. Importantly, these findings are generally smaller than those previously reported and include wider confidence intervals [4], results that are probably important for making decisions regarding the inclusion and use of exercise for reducing resting SBP and DBP in older adults. However, while the overall findings of the current study are generally smaller than the original meta-analysis [4], the results for SBP as a result of resistance training using the IVhet model were larger in the current versus original meta-analysis (-7.0 versus -5.5 mmHg) [4]. One possible explanation for this discrepancy may be the susceptibility of the random-effects model to the positive asymmetry observed (Figure 1) and spuriously smaller findings as a result of such [10]. More broadly, the findings for all outcomes in the current investigation reinforce the susceptibility of the random-effects model to small-study effects [10].

While the reductions are generally smaller than those previously reported, they not only were statistically significant, but also appear to be practically important at the population level, especially with respect to the 4.7 to 7.0 mmHg reductions observed for resting SBP. For example, at the population level, a reduction of 5 mmHg in resting SBP has been associated with a 9%, 14%, and 7% reduction in coronary heart disease, stroke, and all-cause mortality, respectively[11]. Finally, these findings are similar to previous randomized controlled trials and meta-analyses irrespective of age and baseline resting blood pressure values [12].

The major strength of this study was the use of the IVhet model to provide more robust information regarding the effects of aerobic, resistance, and combined aerobic and resistance exercise on resting SBP and DBP in older adults [5]. An additional strength was the use of the recently developed Doi plot and LFK index to provide more robust information regarding potential small-study effects [8]. However, potential limitations exist. First, similar to all aggregate data meta-analyses, the potential for ecological fallacy, specifically Simpson's paradox, exists [13]. Second, the statistical heterogeneity observed warrants further investigation regarding potential predictors associated with changes in resting SBP and DBP among older adults. Third, in the original meta-analysis [4], the investigators stratified their results by length of training while the current meta-analysis avoided such. However, given the lack of justification for these previously used cutpoints as well as the fact that such stratification made little difference in the overall results, this does not appear to be a major limitation of the current investigation [4]. Fourth, the major asymmetry observed for both SBP and DBP as a result of resistance exercise warrants caution in the interpretation of results. Finally, the exact dose-response effects of exercise on resting SBP and DBP remain elusive.

## 5. Conclusions

The overall findings of this brief report suggest that, with the exception of changes in DBP as a result of resistance training, exercise (aerobic, resistance, and combined aerobic and resistance) reduces resting SBP and DBP in older adults. These findings provide important information when considering exercise for the prevention and treatment of raised SBP and DBP in older adults.

## Figures and Tables

**Table 1:** Summary of change outcome differences for resting systolic and diastolic blood pressure using the IVhet model.

Variable	ES(n)	$\overset{-}{X}$ (95% CI)	p	Q(p)	*I* ^*2*^ (95% CI)
Aerobic					
(i) SBP (mmHg)	25	-4.7 (-7.7, -1.8)	0.001^*∗*^	110.0 (<0.001)^*∗∗*^	78.2 (68.3, 85.0)
(ii) DBP (mmHg)	26	-2.0 (-3.1, -0.9)	<0.001^*∗*^	64.5 (<0.001)^*∗∗*^	61.2 (40.6, 74.7)
Resistance					
(i) SBP (mmHg)	13	-7.0 (-10.5, -3.4)	<0.001^*∗*^	40.9 (<0.001)^*∗∗*^	70.7 (48.3, 83.3)
(ii) DBP (mmHg)	13	-1.2 (-2.7, 0.3)	0.12	26.0 (0.01)^*∗∗*^	53.9 (13.7, 75.4)
Both					
(i) SBP (mmHg)	13	-5.5 (-8.3, -2.7)	<0.001^*∗*^	44.9 (<0.001)^*∗∗*^	73.3 (53.5, 84.6)
(ii) DBP (mmHg)	13	-3.7 (-4.8, -2.7)	<0.001^*∗*^	21.3 (0.05)^*∗∗*^	43.8 (0, 70.6)

Notes: results based on the change outcome differences between exercise and control groups; SBP, systolic blood pressure; DBP, diastolic blood pressure; Both represents those who participated in a combined program of aerobic and resistance training; ES(n), number of effect sizes; $\overset{-}{X}$ (95% CI), mean and 95% confidence interval; p, alpha value for changes in blood pressure; Q(p), Cochran's Q statistic for heterogeneity and alpha value for Q; *I*^*2*^ (95% CI), inconsistency statistic and 95% confidence interval; ^*∗*^, statistically significant at an alpha value of ≤ 0.05; ^*∗∗*^, statistically significant at an alpha value of ≤ 0.10.

**Table 2:** Summary of Doi plot results for asymmetry, i.e., small study effects, based on changes in resting systolic and diastolic blood pressure.

Variable	ES(n)	LFK Index	Asymmetry
Aerobic			
(i) SBP (mmHg)	25	-0.32	None
(ii) DBP (mmHg)	26	-0.89	None
Resistance			
(i) SBP (mmHg)	13	2.02	Major
(ii) DBP (mmHg)	13	-3.21	Major
Both			
(i) SBP (mmHg)	13	0.06	None
(ii) DBP (mmHg)	13	0.84	None

Notes: SBP, systolic blood pressure; DBP, diastolic blood pressure; Both, represents those who participated in a combined program of aerobic and resistance training; LFK index, Luis Furuya-Kanamori Index.

## Data Availability

The data used to support the findings of this study are available from the corresponding author upon request.

## Supplementary Materials

Supplementary File 1 Forest plot for changes in resting SBP in mmHg as a result of aerobic exercise.

Supplementary File 2 Forest plot for changes in resting DBP in mmHg as a result of aerobic exercise.

Supplementary File 3 Forest plot for changes in resting SBP in mmHg as a result of resistance exercise.

Supplementary File 4 Forest plot for changes in resting DBP in mmHg as a result of resistance exercise.

Supplementary File 5 Forest plot for changes in resting SBP in mmHg as a result of combined aerobic and resistance exercise.

Supplementary File 6 Forest plot for changes in resting DBP in mmHg as a result of combined aerobic and resistance exercise.

Supplementary File 7 Doi plot for asymmetry, i.e., small-study effects, for changes in resting SBP as a result of aerobic exercise.

Supplementary File 8 Doi plot for asymmetry, i.e., small-study effects, for changes in resting DBP as a result of aerobic exercise.

Supplementary File 9 Doi plot for asymmetry, i.e., small-study effects, for changes in resting DBP as a result of resistance exercise.

Supplementary File 10 Doi plot for asymmetry, i.e., small-study effects, for changes in resting SBP as a result of combined aerobic and resistance exercise.

Supplementary File 11 Doi plot for asymmetry, i.e., small-study effects, for changes in resting DBP as a result of combined aerobic and resistance exercise.
