# Supplementary material for: Brief Report: Exercise and Blood Pressure in Older Adults—An Updated Look
Source: Int J Hypertens. 2018 Oct 29;2018:6548659. doi: 10.1155/2018/6548659 (PMC6232798; doi:10.1155/2018/6548659)
Supplement: Supplementary File 6 — Forest plot for changes in resting DBP in mmHg as a result of combined aerobic and resistance exercise. [file 6548659.f6.docx]

 Supplementary file 6. Forest plot for changes in resting DBP in mmHg as a result of combined aerobic and resistance exercise.
