# Supplementary material for: Brief Report: Exercise and Blood Pressure in Older Adults—An Updated Look
Source: Int J Hypertens. 2018 Oct 29;2018:6548659. doi: 10.1155/2018/6548659 (PMC6232798; doi:10.1155/2018/6548659)
Supplement: Supplementary File 9 — Doi plot for asymmetry, i.e., small-study effects, for changes in resting DBP as a result of resistance exercise. [file 6548659.f9.docx]

Supplementary file 9. Doi plot for asymmetry, i.e., small study effects, for changes in resting DBP as a result of resistance exercise.
